# Prevalence of vestibular disease in France: analysis of prescription data from a national health insurance database

**DOI:** 10.1007/s00415-024-12423-z

**Published:** 2024-05-10

**Authors:** Eva Grill, Vincent Darrouzet, Ulrich Mansmann, Christian Chabbert

**Affiliations:** 1 Institute for Medical Information Processing, Biometry and Epidemiology, Faculty of Medicine, Ludwig-Maximilians University Muenchen, Marchioninistr. 15, 81377 Munich, Germany; 2 German Centre for Vertigo and Balance Disorders, Faculty of Medicine, Ludwig-Maximilians University Muenchen, Munich, Germany; 3 Otolaryngology, Head and Neck Surgery, University of Bordeaux, Bordeaux, France; 4 Research Centre in Psychology and Neuroscience UMR7077, CNRS-Aix Marseille University, Marseille, France; 5 Research Group on Vestibular Pathophysiology; Unit GDR2074 CNRS, Marseille, France

**Keywords:** Vestibular disorders, Vestibular pathologies, Vertigo, Dizziness, Menière’s disease betahistine

## Abstract

Vestibular problems are frequent reasons for primary care consultations. However, there is considerable uncertainty about the prevalence and cost of vestibular disorders. Despite ambiguous effectiveness data, the histamine analogue betahistine is widely and almost exclusively used for treatment of vertigo. Prescription of betahistine can, therefore, be used as a proxy estimate for prevalence. We used openly available claims data from the French health insurance data warehouse, defining annual prevalence of vestibular disease as the number of people who received at least one betahistine prescription that year. Dosage and pack size of each prescribed formulation were extracted to calculate the sum of betahistine in mg and the Defined Daily Dose (DDD) for age and sex strata and in total. To estimate the relative impact of one landmark trial, the BEMED study, we compared prescriptions from the years 2014/2015 to prescriptions in 2019/2022. A total of 735,121 (2014), 694,705 (2015), 614,431 (2019), and 562,476 (2022) persons filled in a prescription of betahistine. Patients were predominantly older and female. Average amount dispensed per year and per person increased from 4422.54 mg during the pre-BEMED period to 4736.90 mg during the post-BEMED period. DDD decreased from 130 Mio per year in 2014/2015 to 116 Mio per year in 2019/2022. Total costs for betahistine decreased by 42% from 21,615,037 Euro in 2014 to 12,894,249 Euro in 2022. Vestibular disease is frequent in France and has a relevant impact on population health. Despite conflicting clinical evidence, betahistine continues to be prescribed widely in medical practice.

## Introduction

In most industrialized countries, vertigo, dizziness, and balance problems are among the most frequent reasons for primary care consultations. Among the recurrent vestibular disorders that cause vertigo, Benign paroxysmal positional vertigo, Menière’s Disease (MD), and Vestibular Migraine account for about 50% of all cases presenting with vertigo. One-year prevalence of vestibular disease has been estimated at 5% by a representative survey in Germany [1]. Vestibular disease may be severely disabling and account for a considerable burden of disease and disability [2–5]. A systematic review of international studies about primary care utilization found that between 1 and 8% of all listed patients had contacted a physician at least once because of vertigo or dizziness [6]. For the United States of America, an average annual total of 48.1 billion USD direct costs for the years 2007 to 2015 were estimated [7], while another analysis of 2018 medical claims data reported an incremental increase of direct costs of 60 billion USD from patients diagnosed with episodic recurrent vestibular vertigo [8]. Total annual direct and indirect costs of MD alone for the UK were between 541.30 million and 608.70 million pound per year [9].

Yet, there is considerable uncertainty around the data reported on prevalence, incidence, and costs of vestibular disease. Large population-based representative cohort studies hardly ever include a detailed workup of vestibular and balance functions. Self-reported occurrence of vertigo or dizziness is not specific for vestibular disease, and patient descriptions may be unclear, inconsistent, and unreliable [10]. The second obvious place for representative data, medical claims data, has limited diagnostic validity, because vestibular disease is not well represented in the ICD-10; therefore, vestibular diagnoses in claims data tend to reflect these inaccuracies of the ICD system [11].

As a potential solution, information on medication from insurance claims data has successfully been used to estimate the prevalence of chronic disease [12]. Thus, it may also be possible to estimate the prevalence of vestibular disease from prescription data. Betahistine is a histamine analogue that is widely [13] and almost exclusively used for treatment of vertigo in general, and specifically as a maintenance medication for the prevention of MD attacks [14]. Using evidence from randomized-controlled trials, effectiveness of betahistine at currently recommended dosage regimes in Menière’s disease seems to be weak [15] including, e.g., one clinical trial that could show no difference to placebo, the Medical Treatment of Menière’s Disease with Betahistine (BEMED) trial [16]. Betahistine at doses higher than the recommended dose was effective in relieving the symptoms in unilateral vestibulopathy [17]. A meta-analysis of 17 studies found a reduction of symptoms in patients with vertigo of different origins; however, risk of bias and heterogeneity of the included studies were high [18].

Betahistine continues to be prescribed, as shown in a recent study from the United Kingdom that compared prescriptions before and after 2016, the year of the BEMED publication [19]. Prescriptions of betahistine can therefore be a good indicator for the prevalence of vestibular disease, because betahistine is not regularly prescribed for other diagnoses. As France has an almost complete coverage of health insurance, we are using data from the French health insurance reporting system to estimate the annual prevalence of vestibular disease by the number of betahistine prescriptions. We were specifically interested in the amount of betahistine substance being prescribed per year, and in the sociodemographic characteristics of the patients who fill in betahistine prescriptions. Additionally, we wanted to investigate if there was any change in prescription practice following the BEMED trial in 2016.

## Methods

### Data sources

In France, health insurance is universally provided as part of the social security system. The statutory health insurance covers most of the costs of medical treatment including medication. Data on medication dispensed in France and fully or partially reimbursed are collected by the French health insurance data warehouse (SNDS) and openly available in aggregated form for download. Data from the SNDS cover almost all beneficiaries of the French health insurance, i.e., 99% of the French population [20]. Betahistine is available only on prescription from retail pharmacies in France.

### Variables

Data from the public aggregated version of the SNDS include information on the hierarchical anatomical therapeutic chemical (ATC) classification codes up to the chemical substance (5th level classification), the unique identifier for all drug presentations in France, the Code Identification Spécialité Pharmaceutique (CIP13), the CIP13 label containing dosage and pack size, the number of packs, the drug costs in Euro, and the amount of costs reimbursed. Costs are prices charged by pharmacies and do not include any costs for acquisition or administration. Sociodemographic information on patients is given on an aggregated level to preserve data protection, namely age in three brackets (0–19 years, 20–59 years, 60+ years), and gender. To avoid de-anonymisation, sociodemographic information is only given for strata with at least ten persons. This includes a total of 38 dose forms that were dispensed very infrequently, which might result in small discrepancies of our results to any summary measures reported elsewhere.

### Statistical analyses

The ATC code N07CA01 was used to extract the prescriptions of betahistine in all trademark and generic formulations. We defined annual prevalence of vestibular disease as the number of people who received at least one betahistine prescription that year. From the CIP13 label, dosage of betahistine and pack size of each formulation were extracted to calculate the sum of prescribed betahistine in mg for each stratum and in total. To give an example, the dose form and package unit “BETAHISTINE ACCORD 08 MG CPR 30” would yield the information that each tablet contains 8 mg of betahistine and each package contains 30 tablets which adds a total of 240 mg betahistine per prescription. Total amount of betahistine in mg per person was then calculated by multiplying the package content in mg by the number of packages of the respective dose form and by dividing the sum of prescribed betahistine per stratum by the number of consumers of each stratum and in total. Defined Daily Dose (DDD) was calculated based on the standard dose of active substance of 24 mg. This dose is not equal to the actual prescribed or effective dose but serves as a standardization method for comparisons.

To compare prescriptions and dosage before and after the BEMED trial, we defined betahistine prescriptions from 2014 and 2015 as pre-BEMED and from 2019 and 2022 as post-BEMED. We hypothesized that prescriptions during the pandemic years 2020 and 2021 would differ from the previous years due to reasons unrelated to our objectives.

SAS (SAS Institute, version 9.4, NC, USA) and Excel Power Pivot (Microsoft Corp.) were used for all analyses.

## Results

A total of 735,121 (2014), 694,705 (2015), 614,431 (2019), and 562,476 (2022) persons received and filled in a prescription of betahistine. Table 1 shows the total amount of mg betahistine and number of packages dispensed, the Defined Daily Dose (DDD), and the total amount of costs and the costs reimbursed, stratified by sociodemographic characteristics and year.

**Table 1:** Prescriptions of betahistine in France

			Persons		Total mg betahistine prescribed		Prescriptions (packages)		Tablets		Dosage per person^a^	Costs reimbursed	Cost basis	DDD
			*n*	%	*n*	%	*n*	%	*n*	%	mg	Euro	Euro	mg
Year		2014	735,121		3,134,345,280		2,820,669		175,520,175		4263.71	8,161,382.03	21,615,036.51	130,597,720
		2015	694,705		3,114,186,240		2,780,183		172,506,630		4482.75	6,725,259.78	17,391,851.91	129,757,760
		2019	614,431		2,838,435,120		2,448,591		150,419,055		4619.62	5,496,010.17	14,104,528.74	118,268,130
		2022	562,476		2,736,388,320		2,324,386		141,516,435		4864.90	5,116,627.98	12,894,248.82	114,016,180
Period		2014/2015	1,429,826	55	6,323,460,480	53	5,600,852	54	352,440,630	55	4422.54	14,886,641.81	39,006,888.42	130,177,740
		2019/2022	1,176,907	45	5,574,891,120	47	4,772,977	46	291,938,310	45	4736.90	10,612,638.15	26,998,777.56	116,142,155
Age	2014	0–19	5000	1	6,843,600	0	6361	0	390,405	0	1368.72	14,802.47	47,957.12	285,150
		20–59	276,574	38	775,919,520	25	665,413	24	40,666,485	23	2805.47	1,808,051.35	5,191,705.36	32,329,980
		60+	450,519	61	2,345,608,080	75	2,143,609	76	134,135,625	76	5206.46	6,323,085.97	16,334,688.60	97,733,670
		Undisclosed	3028	0	5,974,080	0	5286	0	327,660	0	1972.95	15,442.24	40,685.43	248,920
	2015	0–19	4517	1	6,465,120	0	5960	0	364,320	0	1431.29	11,489.84	36,809.07	269,380
		20–59	256,730	37	743,673,840	24	632,396	23	38,614,215	22	2896.72	1,396,369.02	3,992,046.67	30,986,410
		60+	432,086	62	2,360,995,200	76	2,139,191	77	133,368,675	77	5464.18	531.019.36	13,346,455.41	98,374,800
		Undisclosed	1372	0	3,052,080	0	2636	0	159,420	0	2224.55	6381.56	16,540.76	127,170
	2019	0–19	4459	1	6,861,360	0	6076	0	372,540	0	1538.77	10,864.09	34,442.05	285,890
		20–59	223,493	36	688,119,120	24	567,063	23	34,418,610	23	3078.93	1,169,950.62	3298.419.55	28,671,630
		60+	386,146	63	2,142,027,600	75	1,874,303	77	115,561,095	77	5547.20	4,311,976.65	10,764,860.02	89,251,150
		Undisclosed	333	0	1,427,040	0	1149	0	66,810	0	4285.41	3218.81	6807.12	59,460
	2022	0–19	4098	1	6,393,840	0	5714	0	351,030	0	1560.23	9840.52	31,059.25	266,410
		20–59	196,263	35	647,968,800	24	526,807	23	31,827,855	22	3301.53	1,057,985.16	2,958,189.39	26,998,700
		60+	361,913	64	2,081,060,160	76	1,791,119	77	109,292,790	77	5750.17	4,046,556.28	9,900,708.10	86,710,840
		Undisclosed	202	0	965,520	0	746	0	44,760	0	4779.80	2246.02	4292.08	40,230
Gender	2014	Male	217,652	30	896,227,920	29	794,337	28	49,220,460	28	4117.71	2,439,951.68	6,108,519.76	37,342,830
		Female	515,161	70	2,234,926,560	71	2,023,292	72	126,107,625	72	4338.31	5,713,179.98	15,483,862.43	93,121,940
		Undisclosed	2308	0	3,190,800	0	3040	0	192,090	0	1382.50	8250.37	22,654.32	132,950
	2015	Male	207,908	30	908,433,840	2	799,185	29	49,405,410	29	4369.40	2,050,000.95	5,003,630.73	37,851,410
		Female	486,155	70	2,204,874,480	71	1,980,197	71	123,050,640	71	4535.33	4,673,612.08	12,383,234.48	91,869,770
		Undisclosed	642	0	877,920	0	801	0	50,580	0	1367.48	1646.75	4986.70	36,580
	2019	Male	185,497	30	840,929,040	30	713,011	29	43,666,275	29	4533.38	1,701,929.41	4,121,609.28	35,038,230
		Female	428,871	70	1,997,448,480	70	1,735,485	71	106,746,000	71	4657.46	3793.837.07	9,982,361.52	83,225,280
		Undisclosed	63	0	110,880	0	95	0	6780	0	1760.00	243.69	557.94	4620
	2022	Male	169,512	30	811,044,240	30	677,109	29	41,129,190	29	4784.58	1575.640.58	3,772,543.56	33,793,510
		Female	392,908	70	1,925,055,360	70	1,647,059	71	100,373,595	71	4899.51	3540.388.68	912.456.44	80,210,640
		Undisclosed	56	0	288,720	0	218	0	13,650	0	5155.71	598.72	.248.82	12,030

To avoid de-anonymisation, sociodemographic information is only given for strata with at least ten persons

^a^Mean mg betahistine prescribed per person per year; DDD: Defined Daily Dose (basis 24 mg betahistine)

There was a notable decrease of the number of consumers and of most of the other summary measures in 2019 and 2022 as compared to 2014 and 2015, except for mg betahistine per person. Average amount of betahistine dispensed per year and per person was 4263.71 mg in 2014, 4482.75 mg in 2015, 4619.62 mg in 2019, and 4864.90 mg in 2022, increasing from 4422.54 mg during the pre-BEMED period to 4736.90 mg during the post-BEMED period. DDD decreased from an average of 130 Mio per year in 2014/2015 to an average of 116 Mio per year in 2019/2022. Patients aged 60 and over received the highest amount of betahistine per year in all years, with 5206.46 mg in 2014 to 5750.17 mg in 2022. Mg per person increased in both men and women.

Total costs for betahistine decreased by 42% from 21,615,037 Euro in 2014 to 12,894,249 Euro in 2022, of which less than 50% was reimbursed.

The number of available betahistine dose forms in France decreased from 49 in 2014 to 38 in 2022. Most dose forms were either 8 mg or 24 mg. The percentage of dose forms with 8 mg decreased from 61% in 2014 to 47% in 2022.

## Discussion

Our study on the magnitude of filled in prescriptions found that between 2014 and 2022, 560,000 to 740,000 persons insured by the French statutory health insurance received betahistine. Patients with betahistine were predominantly older and female. While package count, total mg prescribed and DDD decreased when comparing years before 2016 to years after, mg per person per year increased steadily in all age groups.

As 99% of the French population is currently inscribed in the statutory health insurance [20], the number of persons with filled in prescriptions of betahistine corresponds to roughly 0.9% of the population of 67Mio persons contained in the SNDS. This number aligns well with the estimated 1% of annual consultations for vestibular disease from other studies [6], albeit at the lower end. The total annual number of 142 Mio prescribed tablets found in our study for 2022 also approximates the monthly 11 Mio tablets prescribed in the UK for an insured population of 57 Mio [19]. We are likely to underestimate the true prevalence of vestibular disease, because not all patients who seek consultation for vestibular symptoms will receive a prescription of betahistine, but the magnitudes we found are in the expected range of persons with moderate-to-severe vertigo.

While betahistine is mainly licenced for Menière’s disease (MD), our estimates of prescriptions largely exceed the prevalence of MD of 40 to 200 per 100,000 in the general population estimated by other studies [21–23]. Partly, this can be explained by the tendency to overdiagnose MD in medical practice [21, 23]. Nevertheless, our findings also confirm that betahistine is prescribed not only for MD but also for a wide range of other vestibular diagnoses. These prescription patterns have also been reported elsewhere [19]. Data from Germany show that about 20% of patients with benign paroxysmal positional vertigo, 63% of MD patients, and 26% of vestibular migraine patients received betahistine in primary care before presenting at a tertiary care clinic [24].

According to our data, prescriptions of betahistine in France decreased considerably between 2014 and 2022. There are several potential explanations for this: first, a decrease of vestibular disease in France, second, an improved, more evidence-based prescription practice following the BEMED trial, or third, reasons related to the pharmaceutical market. To start with disease prevalence, it is unlikely that diagnosis or true prevalence of vestibular disease decreased within 8 years by almost 25%. While fluctuations in the prevalence of vestibular disorders in different regions have been reported, there is no clear indication of a consistent increase or decrease in prevalence over the last decade. Second, decline in prescriptions could be the sign of some improvement of prescribing practice. Interestingly, the use of betahistine in MD or more generally in vestibular disease has no strong base of evidence [15, 18]. Likewise, the French Otorhinolaryngology-Head and Neck Surgery Society does not recommend betahistine as a first line treatment of MD [25]. It can be argued that prescriptions did decrease after 2016, probably also following the publication results from the BEMED clinical trial for betahistine in MD in 2016 [16] and a Cochrane review on the utilization of betahistine in vertigo [18]. At the same time, we found that the amount of betahistine dispensed per person increased. Both findings may be a consequence of the discussions around the low bioavailability of betahistine due to the first-pass elimination [26], which may be counteracted by increasing the oral dose. As patient safety precludes a substantial dose increase, and as parenteral application is hardly feasible, the addition of a pharmacological booster such as selegiline is hypothesized to improve the effectiveness of betahistine [27]. This tendency for higher dosage regimes is also reflected in the decreasing market presence of the low dosage forms found in our data. A third reason for the decline in prescriptions is likely to be market-related, as betahistine formulations have been out of stock or difficult to obtain in France for some time.

Prescriptions of betahistine in France are still on a high level. To give an example, the decrease of DDD in France from 131 Mio mg in 2014 to 114 Mio mg in 2022 found in our data is well above the 68 Mio DDD reported in Germany in a population of 74 Mio insured persons [28]. Of note, the German DDD for betahistine in 2022 was higher as compared to 2019 (62 Mio DDD).

Our study has its strengths in the rigorous database of the SNDS that gives access to a complete, reproducible, and unbiased analysis of medications in the French health care system. Limitations include the lack of individual information on patients including detailed diagnoses and prescription trajectories.

In summary, the prescription of betahistine each year seems to be a good indicator for the prevalence of vestibular disease in France. Vestibular disease is frequent in France and has a relevant impact on population health. Despite conflicting clinical evidence, betahistine continues to be prescribed widely in medical practice.

## Data Availability

Data are available at https://assurance-maladie.ameli.fr/etudes-et-donnees/open-medic-depenses-beneficiaires-medicaments.

## Code availability

SAS-Code is available upon request from the corresponding author.
